# Preoperative Risk Factors for Short-Term Postoperative Mortality of Acute Mesenteric Ischemia after Laparotomy: A Systematic Review and Meta-Analysis

**DOI:** 10.1155/2020/1382475

**Published:** 2020-10-05

**Authors:** Wenhan Wu, Jianbo Liu, Zongguang Zhou

**Affiliations:** Institute of Digestive Surgery of Sichuan University, and Department of Gastrointestinal Surgery, West China Hospital, West China School of Medicine, Sichuan University, 610041 Chengdu, Sichuan, China

## Abstract

**Objective:**

Our objective was to comprehensively present the evidence of preoperative risk factors for short-term postoperative mortality of acute mesenteric ischemia after laparotomy.

**Methods:**

PubMed, Embase, and Google Scholar were searched from January 2000 to January 2020. Studies evaluating the postoperative risk factors for short-term postoperative mortality of acute mesenteric ischemia after laparotomy were included. The outcome extracted were patients' demographics, medical history, and preoperative laboratory tests.

**Results:**

Twenty studies (5011 patients) met the inclusion criteria. Studies were of high quality, with a median Newcastle-Ottawa Scale Score of 7. Summary short-term postoperative mortality was 44.38% (range, 18.80%–67.80%). Across included studies, 49 potential risk factors were examined, at least two studies. Meta-analysis of predictors based on more than three studies identified the following preoperative risk factors for higher short-term postoperative mortality risk: old age (odds ratio [OR], 1.90, 95% confidence interval [CI], 1.57–2.30), arterial occlusive mesenteric ischemia versus mesenteric venous thrombosis (OR, 2.45, 95% CI 1.12–5.33), heart failure (OR 1.33, 95% CI 1.03–1.72), renal disorders (OR 1.61, 95% CI 1.24–2.07), and peripheral vascular disease (OR 1.38, 95% CI 1.00–1.91). Nonsurvivors were older (standardized mean difference [SMD], 0.32, 95% CI 0.24–0.40), had higher creatinine levels (SMD 0.50, 95% CI 0.25–0.75), and had lower platelet counts (SMD −0.32, 95% CI −0.50 to −0.14).

**Conclusion:**

The short-term postoperative mortality of acute mesenteric ischemia who underwent laparotomy is still high. A better understanding of these risk factors may help in the early identification of high-risk patients, optimization of surgical procedure, and improvement of perioperative management.

## 1. Introduction

Acute mesenteric ischemia (AMI) is usually a collection for a group of diseases caused by sudden insufficiency of blood to the intestine, including arterial occlusive mesenteric ischemia (AOMI, 65%–75%), mesenteric venous thrombosis (MVT, 5%–15%), and nonocclusive mesenteric ischemia (NOMI, 10%–30%) [1]. Although the annual incidence rate of AMI is 0.09% to 0.2% [1, 2], it is the most common cause of peritonitis in critically ill patients and an indication for emergency bowel resection [3, 4]. Diagnosis of AMI upon admission is now possible using contrast-enhanced computed tomography (CT) [5], but a considerable number of patients developed peritonitis before the mesenteric revascularization [6]. For these patients, exploratory laparotomy, assessing the intestinal viability, reestablishment of blood supply to the ischemic bowel, and removal of the necrotic intestine are a definite treatment and can improve patient outcome. If the nonviable region was not found and resected, it would eventually induce multiple organ dysfunction, which strongly affected the survival of the AMI patients, and the laparotomy allows us to directly assess the intestinal viability.

Prompt laparotomy is of great significance for the survival of critical AMI. However, the postoperative short-term mortality rate is still about 40% [7, 8], which is undoubtedly disturbing. The preoperative risk factors related to the death of AMI after laparotomy remain unclear from the existing studies because of variations in design and predictors investigated in these studies [9–12]. Identifying preoperative patient-related factors, predicting the postoperative mortality may help to identify high-risk patients, redefine the surgical strategy, and provide layered care for each patient. Such knowledge is also critical for patients and family members to understand the natural course of AMI and possible worst endings. However, to the best of our knowledge, no systematic review has been published to summarize the preoperative risk factors for short-term postoperative death of AMI after laparotomy or to indicate consistent and most valuable predictors.

The study aimed to comprehensively review the published literature to identify the preoperative patient-related variables that increased the postoperative short-term mortality risk of AMI after laparotomy.

## 2. Material and Methods

Preferred Reporting Items for systematic review and Meta-Analyses (PRISMA) guidelines were followed (Table S1). This meta-analysis did not involve human subjects and did not require an Institutional review board review.

### 2.1. Literature Search

We conducted literature searches using PubMed, Embase, and Google Scholar form January 2000 up to January 2020; a combination of Medical Subject Headings (Mesh) terms and free words were used to select the search terms, including combinations and variations of the following keywords “mesenteric vascular occlusion” or “mesenteric ischemia” and “prognosis” or “mortality” or “survival” or “death.” For example, the details of the search steps based on PubMed are shown in Table S2. The language of the literature was limited to English. Conference reports were excluded. Only peer-reviewed studies could be included.

### 2.2. Eligibility Criteria

The two investigators (W.-H. W., J.-B. L.) independently screened the literature using defined eligibility criteria. Firstly, irrelevant studies were excluded based on title and abstract alone. Then the abstracts and full texts of potentially relevant research were reviewed by the two investigators. Any disagreement between investigators was resolved through internal discussion to reach consensus. Where possible, the study authors were contacted for more detailed information.

#### 2.2.1. Inclusion Criteria

Inclusion criteria were as follows: studies of patients with AMI that reported comparative data associated with at least one defined preoperative factor for postoperative mortality, the diagnosis of primary AMI being based on medical history, imaging tests, and laparotomy, and randomized controlled trials, cohort studies, and observational case series.

#### 2.2.2. Exclusion Criteria

Exclusion criteria were as follows: editorials, case reports, review studies, and experimental animal articles, studies reporting overall mortality and risk factors only, and no information being available on postoperative mortality and predictors, studies with incomplete data or other studies where data cannot be extracted, and studies following treatment techniques only or concerning new biomarkers.

### 2.3. Data Collection and Data Items

The studies included in the systematic review were analyzed to identify all reported risk factors for death after laparotomy. The defined preoperative variables were then extracted from the included studies. The following data were also extracted from included studies: authors and publication time, study design, number of cases, and statistical methods. The extracted predictors (preoperative risk factors for postoperative mortality of AMI after laparotomy) were patient demographics, medical history, initial symptoms, physical finds, or preoperative routine laboratory tests.

### 2.4. Risk of Bias within Studies and Quality Assessment

Study quality evaluation was analyzed for each article using the Newcastle-Ottawa Scale (NOS) [13]. NOS scores >7 were considered as high-quality studies, and NOS scores of 5–7 were considered as moderate-quality studies. The GRADE (Grading of Recommendations Assessment Development and Evaluation) was adopted to evaluate the quality of evidence on risk factors for the meta-analyses. The software used was GRADEpro GDT [14]. The evidence quality may be rated as very low, low, moderate, or high. Meta-analyses based on randomized controlled trials are usually considered as high-quality evidence, where results based on observational studies are always regarded as low-quality evidence. The degree of evidence may be upgrade or downgrade. The high risk of bias, high degree of inconsistency (*I*^2^ > 75%), indirectness, or risk of publication bias can downgrade the evidence level, which can also be upgraded by the large outcome effect [14]. If ten studies or more are included in the meta-analysis for any risk factor, a funnel plot and egger's test will be used to assess the risk of publication bias for these risk factors.

### 2.5. Statistical Analysis

The above-mentioned preoperative predictors were reported as both categorical and continuous variables, and these were analyzed separately. Odds ratios (ORs) with 95% confidence interval for the categorical variables with uni- and multivariate analyses were extracted from each included study. If the ORs of the univariate analysis results are not specified, the frequencies were used for calculation whenever possible. Crude ORs were then pooled. To determine the association between continuous predictors and short-term postoperative mortality, the mean and standard deviation between survivors and nonsurvivors was compared and pooled using a standardized mean difference (SMD). SMD is a method to evaluate the variable difference between survivors and nonsurvivors adopting the standardized measure. In general, effect sizes of 0.2, 0.4, and 0.8 are considered small, medium, and large, respectively. Although multivariate analysis takes into account the interaction of preoperative risk factors and potential confounding factors, nonsignificant results have not been presented in many studies. Therefore, the significant results in the multivariate analysis were only listed and reported narratively.

Cochrane's *Q* (*χ*^2^) test and the Higgins *I*^2^ test were used to assess heterogeneity between studies. If heterogeneity was present (*Q* test < 0.1 or *I*^2^ ≥ 25%), a random-effects model as described by Der Simonian and Laird was adopted [15]. Otherwise, a fixed-effect model was applied using the inverse variance method. When two or more studies examined the same potential preoperative risk factor (same definition) in a comparable manner, the meta-analysis was undertaken. We provide data on all levels but mainly focus on those meta-analyses with more than three component studies.

All calculations and graphical representations were performed with the “Metafor” package (version 2.1–0) in the *R* statistics software [16].

## 3. Results

### 3.1. Literature Search and Study Characteristics

**Figure 1:**
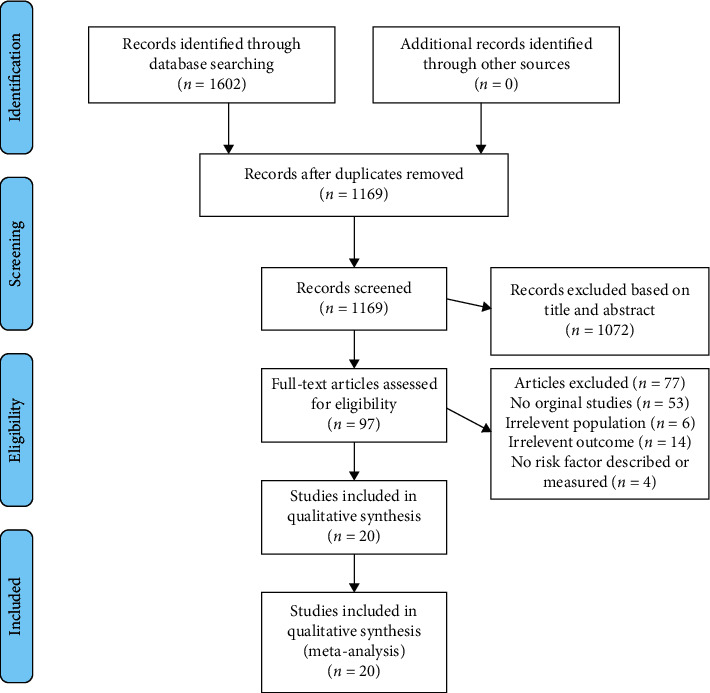
PRISMA flow diagram.

The initial search identified 1602 potentially relevant studies. The majority of studies (*n* = 1505) were excluded based on title or abstract as being irrelevant to the study. The full-text versions were evaluated for the remaining 97 articles (Figure 1). Finally, twenty studies that met the eligibility criteria were identified (Table 1).

A total of 5011 patients were included. All studies described possible preoperative risk factors for short-term postoperative mortality of acute mesenteric ischemia after laparotomy. All included studies were retrospective observational design. The primary outcome measure for each study was mainly 30-day mortality or hospital mortality (Table 1). Eleven studies assessed hospital mortality (median 50.61%) [2, 7, 8, 10, 17–23], eight studies assessed 30-day mortality (median 37.73%) [9, 24–30], and one study assessed the 72-hour mortality (29.1%) after laparotomy [11]. Overall, the median (unweighted) short-term mortality after laparotomy was 44.38% (range, 18.80%–67.80%).

### 3.2. Univariate Analysis of Preoperative Risk Factors

Across the included studies, forty-nine potential clinical factors, including patient demographic, major comorbidities, etiology, initial clinical symptoms, physical findings, computed tomography findings, and laboratory tests, were examined, at least two studies (Table 2).

### 3.3. Multivariate Analysis of Preoperative Risk Factors

Fourteen of the included studies used multivariable models to analyze the risk factors for short-term postoperative mortality of AMI after laparotomy [2, 10, 11, 17, 18, 21, 22, 24–30]. Across these studies, 31 risk factors were found to be statistically significant (Table 3). Of these, only age was significant in more than one study.

### 3.4. Meta-Analysis

Forty-nine preoperative clinical factors had comparable data in at least two studies (same clinical factor, available data). The full details of this meta-analysis are shown in Table 2. Eighteen factors had comparable data in more than three studies, of which eight were found to be significant on meta-analysis. However, for each risk factor, no one included more than ten studies; these numbers were too small so that the test efficacy of funnel plots and egger's test was insufficient [31]. Therefore, funnel plots and Egger's test were not assessed to calculate the risk of bias.

#### 3.4.1. Old Age

**Figure 2:**
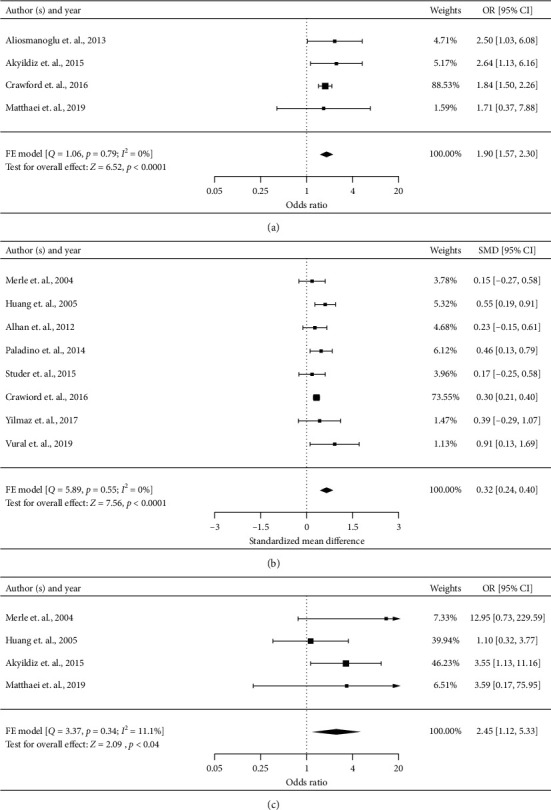
Forest plots for significant preoperative risk factors for short-term postoperative mortality of AMI after laparotomy with data available in at least four studies (demographics and etiology). AOMI: arterial occlusive mesenteric ischemia; FE: fixed effect; MVT: mesenteric venous thrombosis; RE: random effect. (a) Old age (categorical variable), (b) age (continuous variable), and (c) AOMI versus MVT (categorical variable).

Four studies analyzed the predictive value of old age using a cut-off between 60 and 70 for short-term postoperative mortality in patients with AMI [2, 9, 20, 30]. Meta-analysis of these studies (*n* = 2502) showed a significant association of old age and increased short-term postoperative mortality risk of AMI (OR 1.90, 95% CI 1.57–2.30, *P* < 0.0001) (Figure 2(a)). There was no heterogeneity across these studies (*I*^2^ = 0%). The quality of evidence for old age as a risk factor was low based on the GRADE method because of the observational nature of the included studies.

#### 3.4.2. Age

Nine studies compared the age between survivors and nonsurvivors after laparotomy [2, 7, 8, 11, 17, 19, 23, 25, 29]. One study was excluded because the data were not presented with mean ± standard deviation [25]. Meta-analysis of the remaining eight studies (*n* = 2900) indicated that nonsurvivors had a higher age than survivors (SMD 0.32, 95% CI 0.24–0.40, *P* < 0.0001) (Figure 2(b)). There was no heterogeneity across these studies (*I*^2^ = 0%). The quality of evidence for age as a risk factor was low based on the GRADE method because of the observational nature of the included studies.

#### 3.4.3. Arterial Occlusive Mesenteric Ischemia versus Mesenteric Venous Thrombosis

In terms of the etiology of AMI, four studies evaluated the difference of postoperative mortality risk between arterial occlusive mesenteric ischemia (AOMI) and mesenteric venous thrombosis (MVT) [9, 11, 17, 30]. Meta-analysis of these studies (*n* = 379) showed the short-term postoperative risk was higher in patients with AOMI than that in MVT (OR 2.45, 95% CI 1.12–5.33, *P*=0.04) (Figure 2(c)). There was low heterogeneity across these studies (*I*^2^ = 11.1%). Compared with MVT, the quality of evidence for AOMI as a risk factor was moderate based on the GRADE method because of the size of the estimate.

#### 3.4.4. Heart Failure

**Figure 3:**
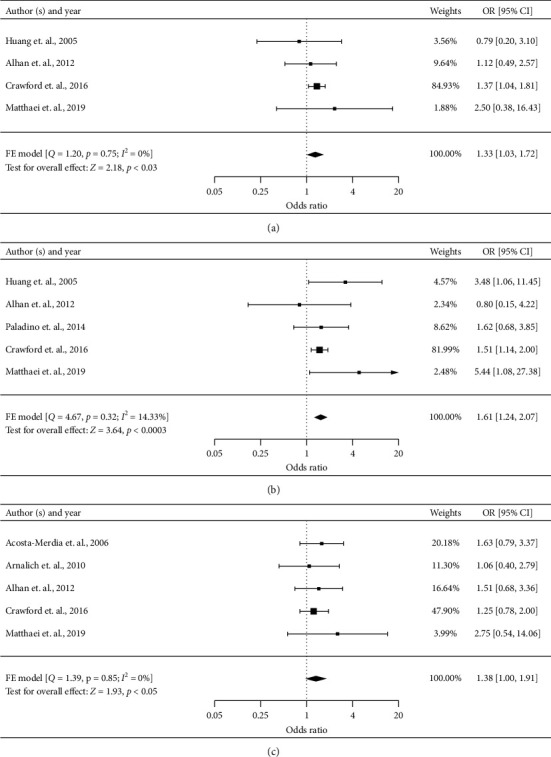
Forest plots for significant preoperative risk factors for short-term postoperative mortality of AMI after laparotomy with data available in at least four studies (comorbidities). Notes: renal disorders include renal failure and chronic renal disease. FE: fixed effect; RE: random effect. (a) Heart failure (categorical variable), (b) renal disorders (categorical variable), and (c) peripheral vascular disease (categorical variable).

Four studies described the influence of heart failure on short-term postoperative mortality [2, 7, 9, 17]. Meta-analysis of four studies (*n* = 2534) showed a significantly higher risk of postoperative mortality in AMI patients with heart failure (OR 1.33, 95% CI 1.03–1.72, *P*=0.03) (Figure 3(a)). There was no heterogeneity across these studies (*I*^2^ = 0%). The quality of evidence for heart failure as a risk factor was low based on the GRADE method because of the observational nature of the included studies.

#### 3.4.5. Renal Disorders

Renal disorders including renal failure and chronic renal disease were examined in five studies [2, 7, 9, 17, 23]. Meta-analysis of these studies (*n* = 2683) showed a significantly higher risk of postoperative mortality in AMI patients with renal disorders (OR 1.61, 95% CI 1.24–2.07, *P*=0.0003) (Figure 3(b)). There was low heterogeneity across these studies (*I*^2^ = 14.33%). The quality of evidence for renal disorders as a risk factor was low based on the GRADE method because of the observational nature of the included studies.

#### 3.4.6. Peripheral Vascular Disease

Five studies evaluated the prognostic value of a previous peripheral vascular disease for short-term postoperative of AMI after laparotomy [2, 7, 9, 10, 25]. Meta-analysis of these studies (*n* = 2641) showed a significantly higher risk of postoperative mortality in AMI patients with previous peripheral vascular disease (OR 1.38, 95% CI 1.00–1.91, *P*=0.05) (Figure 3(c)). There was no heterogeneity across these studies (*I*^2^ = 0%). The quality of evidence for peripheral vascular disease as a risk factor was low based on the GRADE method because of the observational nature of the included studies.

#### 3.4.7. Creatinine

**Figure 4:**
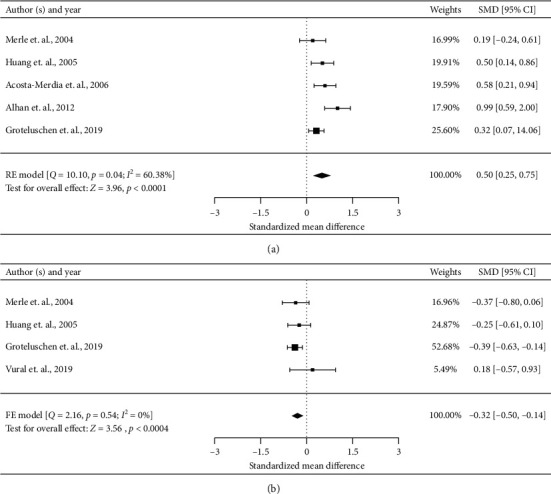
Forest plots for significant preoperative risk factors for short-term postoperative mortality of AMI after laparotomy with data available in at least four studies (laboratory tests). FE: fixed effect; RE: random effect. (a) Creatinine (continuous variable) and (b) platelet (continuous variable).

The level of serum creatinine was compared in six studies [7, 10, 11, 17, 22, 25]. Among these, one had to be excluded because of the unformatted data [25]. Meta-analysis of the remaining five studies (*n* = 768) indicated that nonsurvivors had a higher creatinine than survivors (SMD 0.50, 95% CI 0.25–0.75, *P* < 0.0001) (Figure 4(a)). There was moderate heterogeneity across these studies (*I*^2^ = 60.38%). The quality of evidence for creatinine as a risk factor was moderate based on the GRADE method because of the size of the estimate.

#### 3.4.8. Platelet

The level of platelet was evaluated in four studies [11, 17, 22, 29]. Meta-analysis of four studies (*n* = 566) indicated that nonsurvivors had a lower platelet than survivors (SMD −0.32, 95% CI −0.50 to −0.14, *P*=0.0004) (Figure 4(b)). There was no heterogeneity across these studies (*I*^2^ = 0%). The quality of evidence for platelet as a risk factor was low based on the GRADE method because of the observational nature of the included studies.

### 3.5. Risk of Bias and Quality Assessment

All the included studies were assessed for risk of bias using NOS. The median score for all studies was 7 (range 6–8) (Table 1). Because all included studies were observational design, the quality of evidence in the meta-analyses all started with low quality. Two outcomes were upgraded as a result of the large size of the estimate. For each preoperative risk factor, there was no severe heterogeneity among these studies. Therefore, no evidence grade was downgraded (Table 4).

## 4. Discussion

This study is the first meta-analysis to assess preoperative risk factors for short-term postoperative mortality of AMI after laparotomy, including twenty studies with 5011 patients. AMI is a surgical emergency due to a sudden insufficient supply of blood to the intestine. For patients who highly suspect intestinal necrosis, surgical interventions can reduce mortality [32]. From our research, short-term postoperative mortality of AMI has decreased in the past two decades, but it is still around 40% [33, 34]. Identifying the potential preoperative risk factors could be useful to help guide more personalized perioperative management of AMI which requires laparotomy, vascular treatment, and decisions to escalate or withdraw treatment. Our findings demonstrate that older age, heart failure, renal disorders, peripheral vascular disease, higher creatinine levels, and lower platelet counts are risk factors for outcome. In addition, compared with MVT, the prognosis of patients with AOMI is worse. Although the level of evidence for risk factors was regarded low or moderate, this was not a result of biases across studies, but mostly as a result of the observational nature of the included studies which results in a low-quality starting point of evidence.

The incidence of AMI has increased exponentially with age, and AMI is a more common cause of acute abdomen than appendicitis in patients aged 75 years [35]. The present systematic review indicates that the advanced age is a risk factor for postoperative mortality. Besides, the mean age is significantly higher in nonsurvivors than survivors. There is some explanation as to why a higher age may lead to higher postoperative mortality. The elderly had a higher mortality rate than young patients after emergency surgery, even those who were in a generally good physical condition [36]. Another reason may be related to the delay in diagnosis caused by the more atypical presentations of AMI in the elderly. In general, there are three different aetiological forms of AMI, including AOMI, MVT, and NOMI. There was evidence showing that the outcome of AOMI and NOMI is even worse after surgical treatment based on the literature before 2002 [34]. Our study also confirmed that the short-term postoperative mortality of AOMI is higher than that of MVT. This may be related to the location of the occlusion often occurring at the proximal part of the intestinal vessels leading to more extensive intestinal necrosis and bowel resection [37]. For NOMI patients who underwent laparotomy, there is no significant evidence that the mortality is higher compared with MVT from our study, but the prognosis of NOMI still seemed to be worse. It is worth noting that the effectiveness of therapy for NOMI also depends on the control of the primary underlying disease.

Another feature of AMI is that preexisting comorbidities were common. Although atrial fibrillation and coronary heart disease are associated with the prevalence of AMI, they did not affect postoperative survival. Our study confirmed that heart failure is a risk factor for postoperative death of AMI. In addition, a study has shown that even patients with the least severe heart failure also had higher mortality after surgery [38]. Therefore, when it comes to preoperative cardiac risk assessment of AMI, the stratification and management of heart failure is crucial. Optimizing fluid status before laparotomy may improve the outcome of patients. There is no obvious explanation of why the previous peripheral vascular disease is a risk factor for short-term postoperative mortality of AMI, which may be associated with more mesenteric arterial thrombosis leading to the poor prognosis [39].

In this study, another factor closely related to postoperative survival was kidney conditions. Previous renal disorders or elevated creatinine were predictors of an increased risk of postoperative death. The treatment strategy should not only focus on early surgical intervention. Proper fluid replacement and avoidance of drug toxicity to the kidney were also crucial. For those patients with AMI who already had chronic kidney disease, in order to promote clinical decision making, a prospective cohort study is needed to compare the therapeutic effects of different interventions. Testing interventions to reduce mortality in these patients remain a top priority.

This study also found that nonsurvivors had lower preoperative platelet counts. Hypoxia and hypercapnia of the intestinal mucosa caused by AMI damaged its barrier functional integrity [40]. Bacteria and their toxins were carried through the blood to the whole body, which was the basis of septic shock. Thrombocytopenia is very common in sepsis and is a sensitive marker of disease severity [41]. However, the platelets have multiple physiological roles in AMI. On the one hand, platelets may promote coagulation and inflammation, and, on the other hand, platelets are closely related to the clearness of pathogens. In addition, platelets can protect the integrity of blood vessels. Management platelet levels in patients with AMI during the perioperative period are worth exploring.

Our study also has several limitations. The study was not designed to test a prespecific exposure for postoperative mortality but rather to systematically evaluate reported factors commonly measured on admission in observational studies of AMI patients who underwent laparotomy. Besides, another limitation may be the size of the included studies which is not sufficient to assess preoperative risk factors for AMI of each subtype. Therefore, we should be more cautious in interpreting the results of this meta-analysis.

## 5. Conclusion

In this study, we present a summary and have used meta-analysis to quantify the preoperative risk factor for the short-term mortality of AMI after laparotomy. Creatinine and platelet could be considered as potentially “modifiable,” and others may be used to identify at-risk patients. The preoperative risk factors for short-term postoperative mortality of AMI should be more closely examined to clarify the interaction between the risk factors and each subtype of AMI and eventually form a consensus to improve the prognosis of patients with AMI who require laparotomy.

## Figures and Tables

**Table 1:** Characteristics of studies.

Study	Size, no.	Endpoint	Mortality (%)	NOS score
Kougias et al., 2007, USA	72	30-day mortality	31.9	7
Crawford et al., 2016, USA	2255	Hospital mortality	24.4	6
Alhan et al., 2012, Turkey	107	Hospital mortality	55.1	7
Matthaei et al., 2019, Germany	48	30-day mortality	18.8	8
Edwards et al., 2003, USA	77	Hospital mortality	62.3	6
Arnalich et al., 2010, Spain	99	30-day mortality	46.6	7
Huang et al., 2005, China	124	Hospital mortality	50.0	7
Park et al., 2002, USA	58	30-day mortality	32.8	7
Acosta-Merida et al., 2006, Spain	132	Hospital mortality	65.2	8
Hsu et al., 2006, China	77	30-day mortality	53.2	6
Gupta et al., 2011, USA	861	30-day mortality	27.9	6
Yılmaz et al., 2017, Turkey	34	Hospital mortality	44.1	7
Aliosmanoglu et al., 2013, Turkey	95	Hospital mortality	42.1	8
Marchena-Gomez et al., 2009, Spain	186	Hospital mortality	64.5	6
Groteluschen et al., 2019, Germany	302	Hospital mortality	67.8	7
Vural et al., 2019, Turkey	37	30-day mortality	24.3	6
Akyildiz et al., 2015, Turkey	104	30-day mortality	66.3	7
Merle et al., 2004, France	103	72-hour mortality	29.1	7
Studer et al., 2015, Switzerland	91	Hospital mortality	42.9	7
Paladino et al., 2014, Italy	149	Hospital mortality	38.3	7

NOS, Newcastle-Ottawa Scale.

**Table 2:** Association between clinical characteristics and short-term postoperative mortality.

Factor	Number of studies	Number of patients	OR (95% CI) (nonsurvivors: survivors) or standardized mean difference (nonsurvivors-survivors) of factor^*∗*^	*P* value	Heterogeneity (*I*^2^), %
Demographic					
Age	8	2900	0.32 (0.24–0.40)^*∗*^	<0.0001	0.00
Old age	4	2502	1.90 (1.57–2.30)	<0.0001	0.00
Male sex	11	3126	1.03 (0.87–1.21)	0.75	4.46

Comorbidities					
Coronary heart disease	6	2782	1.14 (0.70–1.88)	0.59	59.24
Atrial fibrillation	4	581	1.42 (0.79–2.55)	0.24	52.01
Heart failure	4	2534	1.33 (1.03–1.72)	0.03	0.00
Hypertension	6	2866	1.19 (0.57–2.48)	0.64	87.56
Atherosclerosis	2	393	0.84 (0.54–1.30)	0.43	0.00
Arrhythmia	2	2379	1.62 (1.33–1.98)	<0.0001	0.00
Previous cardiac disease	3	326	1.80 (0.86–3.73)	0.12	55.84
Diabetes	9	3307	1.51 (0.97–2.36)	0.07	65.92
Chronic lung disease	3	2453	1.34 (1.04–1.73)	0.02	0.00
Renal disorders	5	2683	1.61 (1.24–2.07)	0.0003	14.33
Peripheral vascular diseases	5	2641	1.38 (1.00–1.91)	0.05	0.00
Comorbidity	2	199	3.49 (1.88–6.46)	<0.0001	0.00

Etiology					
AOMI vs MVT	4	379	2.45 (1.12–5.33)	0.04	11.10
NOMI versus AOMI	5	486	1.33 (0.56–3.16)	0.52	56.86
NOMI versus MVT	4	379	2.50 (0.79–7.93)	0.12	27.23

Medications history					
Antiplatelet	5	764	2.23 (0.77–6.44)	0.14	81.15
Anticoagulant therapy	3	525	0.59 (0.19–1.79)	0.35	65.48
Digoxin	2	239	3.77 (2.02–7.02)	<0.0001	0.00

Initial clinical symptoms					
Abdominal pain	4	462	0.71 (0.18–2.81)	0.63	74.02
Abdominal distension	2	231	1.43 (0.76–2.68)	0.27	0.00
Diarrhea	2	231	0.62 (0.31–1.27)	0.19	0.00
Vomiting	2	231	0.59 (0.35–0.99)	0.05	0.00

Physical findings					
Fever	2	239	0.96 (0.44–2.07)	0.91	0.00
Body temperature	2	231	0 (−0.26–0.25)^*∗*^	0.99	0.00
Pulse rate	2	231	0.42 (0.16–0.68)^*∗*^	0.002	6.58
Blood pressure	2	231	−1.00 (−2.18–0.18)^*∗*^	0.1	94.38
Hypotension after admission	2	227	2.86 (1.39–5.91)	0.005	0.00
peritonitis	2	210	1.72 (0.93–3.17)	0.08	0.00
Sepsis	2	227	2.10 (1.16–3.80)	0.01	0.00
Shock	2	239	4.18 (1.99–8.78)	0.0002	0.00

Computed tomography findings					
Bowel-wall thickening	2	426	0.49 (0.24–0.99)	0.05	33.45
Intramural pneumatosis	2	426	3.87 (0.23–63.98)	0.35	84.90

Laboratory tests					
White blood cell	7	917	0.04 (−0.46–0.53)^*∗*^	0.89	91.47
Platelet	4	566	−0.32 (−0.50 to −0.14)^*∗*^	0.0004	0.00
Hemoglobin	3	541	−0.16 (−0.46–0.14)^*∗*^	0.29	60.29
Amylase	3	342	1.24 (−0.22–2.70)^*∗*^	0.1	96.98
AST	2	405	0.60 (0.10–1.10)^*∗*^	0.02	74.99
CPK	3	541	0.59 (0.00–1.17)^*∗*^	0.05	89.02
Lactate	2	405	0.85 (0.58–1.12)^*∗*^	<0.0001	25.49
PH	3	541	−1.11 (−1.67 to −0.55)^*∗*^	<0.0001	86.89
BUN	2	231	0.93 (0.11–1.75)^*∗*^	0.03	88.69
Creatinine	5	768	0.50 (0.25–0.75)^*∗*^	<0.0001	60.38
Bicarbonate	2	239	−2.34 (−5.78 to 1.09)^*∗*^	0.18	98.77
Bilirubin	2	405	0.12 (−0.09 to 0.33)^*∗*^	0.26	0.00
CRP	2	339	0.29 (0.05–0.52)^*∗*^	0.02	0.00
CRP ≥ 100 mg/L	2	350	0.46 (0.29–0.75)	0.002	0.00

^*∗*^Continuous variables compared by standardized mean difference. A negative value indicates mean value was lower in nonsurvivors than survivors. AST: aspartate aminotransferase; AOMI: arterial occlusive mesenteric ischemia; BUN: blood urea nitrogen; CPK: creatine phosphokinase; CRP: C-reactive protein; MVT: mesenteric venous thrombosis; NOMI: nonocclusive mesenteric ischemia.

**Table 3:** Summary of risk factors analyzed in multivariate models.

Risk factors	Study	OR (95% CI)	*P* value
Patient factors			
Age > 60 years	Park et al., 2002	3.0 (1.3–6.9)	0.0093
Age > 65 years	Crawford et al., 2016	1.8 (1.4–2.3)	<0.0001
Age > 65 years	Huang et al., 2005	1.08 (1.01–1.15)	0.02
Age > 70 years	Kougias et al., 2007	3.6 (1.2–4.2)	0.03
Age (for each increase of 1 year)	Gupta et al., 2011	1.04 (1.02–1.06)	<0.05
Age (for each increase of 1 year)	Marchena-Gomez et al., 2009	1.034 (1.003–1.066)	0.031
Age (for each increase of 1 year)	Vural et al., 2019	1.14 (1.005–1.303)	<0.02
ASA class 1^*a*^	Gupta et al., 2011	0.04 (0.004–0.35)	<0.05
ASA class 2^*a*^	Gupta et al., 2011	0.15 (0.06–0.37)	<0.05
ASA class 3^*a*^	Gupta et al., 2011	0.27 (0.13–0.57)	<0.05
ASA class 4^*a*^	Gupta et al., 2011	0.40 (0.19–0.84)	<0.05
Cardiac dysrhythmia	Crawford et al., 2016	1.5 (1.1–1.9)	0.003
Cardiac illness	Acosta-Merida et al., 2006	2.60 (1.02–6.62)	0.045
Chronic kidney disease	Crawford et al., 2016	1.8 (1.4–2.3)	<0.0001
Heart failure	Merle et al., 2004	5.9 (1.1–31.8)	0.029
Hypercoagulability	Crawford et al., 2016	2.6 (1.8–3.7)	<0.0001
Metabolic acidosis	Huang et al., 2005	6.604 (1.804–24.171)	0.01
NOMI versus MVT	Hsu et al., 2006	12.367 (1.450–105.455)	0.021
Peritonitis	Edwards et al., 2003	22.9 (2.3–225.2)	0.007
Preoperative hypotension	Edwards et al., 2003	14.9 (1.4–160.6)	0.026
Previous surgery	Park et al., 2002	2.4 (1.2–4.9)	0.0229
Prolonged symptoms duration	Kougias et al., 2007	4.6 (1.3–5.1)	0.02
Sepsis	Gupta et al., 2011	3.02 (1.33–6.84)	<0.05

Perioperative factors			
Abnormal albumin	Gupta et al., 2011	2.71 (1.32–5.57)	<0.05
AST >200 IU/L	Merle et al., 2004	8.5 (1.7–41.9)	<0.001
Bandemia	Huang et al., 2005	3.894 (1.160–13.074)	0.03
Blood hemoglobin (for each increase of 1 g/dl)	Arnalich et al., 2010	0.24 (0.10–0.40)	0.001
BUN (for each increase of 1 mg/dl)	Huang et al., 2005	7.219 (1.166–44.696)	0.03
Creatinine (for each increase of 1 mg/dl)	Marchena-Gomez et al., 2009	2.137 (1.3–3.6)	0.003
Creatinine level ≥ 2 mg/dl	Akyildiz et al., 2015	2.4	0.04
CRP > 100 mg/L	Groteluschen et al., 2019	1.758 (1.012–3.054)	<0.001
Elevated AST	Huang et al., 2005	4.532 (1.274–16.122)	0.02
Glucose (for each increase of 1 mmol/l)	Arnalich et al., 2010	1.030 (1.01–1.25)	0.001
Lactate > 3 mmol/L	Groteluschen et al., 2019	2.717 (1.561–4.729)	<0.001
Lactate > 5 mmol/L	Merle et al., 2004	5.5 (1.2–24.5)	0.014
PCT > 40 ng/L	Merle et al., 2004	7.4 (1.3–39.2)	0.006
Urea levels (for each increase of 1 mmol/l)	Acosta-Merida et al., 2006	33.89 (5.07–226.51)	<0.001

^*a*^ASA class 5. ASA, American Society of Anesthesiologists Physical Status Classification; AST, aspartate aminotransferase; BUN, blood urea nitrogen; CRP, C-reactive protein; MVT, mesenteric venous thrombosis; NOMI, nonocclusive mesenteric ischemia; PCT, procalcitonin.

**Table 4:** Summary finds of preoperative risk factors eligible for meta-analysis.

Risk factor	Number of patients/studies	Regarded as a risk factor	Pooled odds ratio/standardized mean difference^*∗*^	Heterogeneity (*I*^2^), %	Quality of evidence (GRADE)
Advanced age	2502/4	Yes	1.90 (1.57–2.30)	0	Low
Age	2751/7	Yes	0.31 (0.22–0.40)^*∗*^	0	Low
AOMI versus MVT	379/4	Yes	2.45 (1.12–5.33)	11.1	Moderate
Heart failure	2534/4	Yes	1.33 (1.03–1.72)	0	Low
Renal disorders	2534/4	Yes	1.93 (1.03–3.62)	35.74	Low
Peripheral vascular disease	2641/5	Yes	1.38 (1.00–1.91)	0	Low
Creatinine	768/5	Yes	0.50 (0.25–0.75)^*∗*^	60.38	Moderate
Platelet	566/4	Yes	−0.32 (−0.50 to −0.14)^*∗*^	0	Low

^*∗*^Continuous variables compared by standardized mean difference. A negative value indicates mean value was lower in nonsurvivors than survivors. Abbreviations: AOMI: arterial occlusive mesenteric ischemia. Notes: MVT, mesenteric venous thrombosis.

## Additional Points

(1) The short-term postoperative mortality of AMI after laparotomy is still high. This study systematically summarized the risk factors associated with short-term postoperative mortality in terms of patients' demographic characteristics, comorbidities, clinical symptoms, physical findings, and preoperative laboratory tests. (2) Old age, heart failure, or renal disorders are detrimental to postoperative survival. Early identification of high-risk patients with AMI can help improve postoperative survival. Optimizing the management of renal function and platelets during the perioperative may help to further reduce postoperative mortality.

## Supplementary Materials

Supplementary Materials Table S1: PRISMA stands for Preferred Reporting Items for Systematic Reviews and Meta-Analyses. It is an evidence-based minimum set of items for reporting in systematic reviews and meta-analyses. Table S2 gives an example, using PubMed to search literature. It contains key search terms and search logic expressions.
