# Supplementary material for: Preoperative Risk Factors for Short-Term Postoperative Mortality of Acute Mesenteric Ischemia after Laparotomy: A Systematic Review and Meta-Analysis
Source: Emerg Med Int. 2020 Oct 5;2020:1382475. doi: 10.1155/2020/1382475 (PMC7556094; doi:10.1155/2020/1382475)
Supplement: Supplementary Materials — Table S1: PRISMA stands for Preferred Reporting Items for Systematic Reviews and Meta-Analyses. It is an evidence-based minimum set of items for reporting in systematic reviews and meta-analyses. Table S2 gives an example, using PubMed to search literature. It contains key search terms and search logic expressions. [file 1382475.f1.zip › 1382475.f1/Table S2 Example search..docx]

Table S2 Example search: Pubmed.

| #1 | mesenteric vascular occlusion【Mesh Terms】 |
| --- | --- |
| #2 | mesenteric or intestinal or bowel |
| #3 | ischemia or ischaemia |
| #4 | #2 and #3 |
| #5 | #1 or #4 |
| #6 | prognos* or perdict* or death or mortality or survival or outcome |
| #7 | #5 and #6 |
